# Anatomical and Functional MRI Changes after One Year of Auditory Rehabilitation with Hearing Aids

**DOI:** 10.1155/2018/9303674

**Published:** 2018-09-10

**Authors:** M. R. Pereira-Jorge, K. C. Andrade, F. X. Palhano-Fontes, P. R. B. Diniz, M. Sturzbecher, A. C. Santos, D. B. Araujo

**Affiliations:** ^1^Department of Neuroscience and Behavior, University of São Paulo, Ribeirao Preto, SP, Brazil; ^2^Brain Institute/Onofre Lopes University Hospital, Federal University of Rio Grande do Norte (UFRN), Natal, RN, Brazil; ^3^Department of Internal Medicine, Federal University of Pernambuco, Recife, PE, Brazil; ^4^Department of Internal Medicine, University of São Paulo, Ribeirao Preto, SP, Brazil

## Abstract

Hearing aids (HAs) are an effective strategy for auditory rehabilitation in patients with peripheral hearing deficits. Yet, the neurophysiological mechanisms behind HA use are still unclear. Thus far, most studies have focused on changes in the auditory system, although it is expected that hearing deficits affect a number of cognitive systems, notably speech. In the present study, we used audiometric evaluations in 14 patients with bilateral hearing loss before and after one year of continuous HA use and functional magnetic resonance imaging (fMRI) and cortical thickness analysis in 12 and 10 of them compared with a normal hearing control group. Prior to HA fitting, fMRI activity was found reduced in the auditory and language systems and increased in visual and frontal areas, expanding to multimodal integration cortices, such as the superior temporal gyrus, intraparietal sulcus, and insula. One year after rehabilitation with HA, significant audiometric improvement was observed, especially in free-field Speech Reception Threshold (SRT) test and functional gain, a measure of HA efficiency. HA use increased fMRI activity in the auditory and language cortices and multimodal integration areas. Individual fMRI signal changes from all these areas were positively correlated with individual SRT changes. Before rehabilitation, cortical thickness was increased in parts of the prefrontal cortex, precuneus, fusiform gyrus, and middle temporal gyrus. It was reduced in the insula, supramarginal gyrus, medial temporal gyrus, occipital cortex, posterior cingulate cortex, and claustrum. After HA use, increased cortical thickness was observed in multimodal integration regions, particularly the very caudal end of the superior temporal sulcus, the angular gyrus, and the inferior parietal gyrus/superior temporal gyrus/insula. Our data provide the first evidence that one year of HA use is related to functional and anatomical brain changes, notably in auditory and language systems, extending to multimodal cortices.

## 1. Introduction

Peripheral hearing deficits have a profound impact on the central auditory system, hampering individual communication and social interaction [1]. Individuals with hearing impairment can benefit from rehabilitation with cochlear implant (CI) and acoustic hearing aid (HA) devices. In both cases, patients experience significant improvement in their general condition, including cognitive abilities such as memory and language comprehension [2, 3].

Little is known, however, about neurophysiological mechanisms underlying these beneficial changes, and most knowledge on the topic is still based on animal models. Lesions to different segments of the auditory system are associated with specific changes in the neuronal representation of sound stimuli in cats [4], monkeys [5], mice [6], birds [7], and rabbits [8]. Furthermore, molecular and electrophysiological evidences show that rehabilitation with CI, for instance, leads to changes in the auditory system [8, 9].

In humans, advances in neuroimaging have expanded considerably the exploration of the auditory system, both in normal hearing subjects [10] and in patients with hearing impairment [11, 12]. Positron emission tomography (PET) and functional MRI (fMRI) have already found consistent reduced activity of the auditory cortex in patients with hearing deficits [13, 14], which is at least partially recovered with CI and HA [12, 14, 15].

Only very few studies used neuroimaging to probe the impact of auditory rehabilitation over higher cognitive functions, and most of them have focused on language cortices, particularly Wernicke's area (Brodmann area—BA22) [12, 16]. In general, auditory deprivation leads to decreased activation of this area, which is recovered at least partially by rehabilitation, for instance, with CI [17]. It has been regarded as a fact that the use of hearing devices allows access to the auditory information to language centers, therefore leading to increased activity of this area. However, to our knowledge, these are still no solid evidence suggesting that this is the case or if there are other mechanisms involved. Thus, the first aim of this longitudinal study is to investigate the impact of HA use over audiometric scales, anatomical and functional MRI, and their correlations.

Furthermore, it is well known that the integration of auditory and visual information greatly improves the ability of language comprehension [18]. In fact, patients with hearing deficits often exhibit increased activity in areas related to visual functions, during auditory stimulation [19, 20]. Therefore, we also aimed to deeply explore brain areas involved in multimodal integration, such as the superior temporal sulcus (STS), the middle intraparietal sulcus (IT, BA40), the inferior frontal gyrus (IFG, BA44, BA45, and BA47), and the insula (BA13). The second objective of this study was to explore effects of auditory deprivation and recovery in sensory integration systems, for aurally delivered stimuli.

## 2. Material and Methods

This work was approved by the Ethics and Research Committee of the University of São Paulo, Ribeirao Preto School of Medicine (no. 2413/2007). Written informed consent was obtained from all participants. The data that support the findings of this study are available from the corresponding author upon request.

### 2.1. Subjects

Two groups participated in the current study: 14 postlingual deaf patients (P) with sensorineural hearing loss (5 women, age = 51.29 ± 18.8 years) and 11 normal hearing control group (CG) (5 women, age = 46.54 ± 19.88 years). At the time of recruitment, all patients had mild to severe bilateral sensorineural hearing loss and were referred to us by an otorhinolaryngologist for HA use (see Suppl.  Table 1 for clinical details).

### 2.2. Audiometric Evaluation and Hearing Aid

The HAs used were manufactured by Widex (Lynge, Denmark). Four patients were fitted with completely in the canal (CIC) HA, and ten patients were fitted with intracanal (ITC) HA, with digital processing and compression (Suppl.  Table 1). During the first two months of HA fitting, patients were evaluated weekly. After acclimatization, all patients were asked to use the HA for at least 10 hours a day.

Audiological evaluation followed the Brazilian protocol and occurred twice: right before HA fitting and right after one year of continuous HA use. All patients underwent pure tone audiometry tests by air and bone in an acoustic cabin, with headphones, for the following frequencies: 250 Hz, 500 Hz, 1000 Hz, 2000 Hz, 3000 Hz, 4000 Hz, 6000 Hz, and 8000 Hz. The pure tone auditory threshold was defined as the minimum level of sound intensity necessary for the pure tone, at each frequency, to be perceived. Patients were instructed to press a button every time they heard a sound (whistle) in the ear being tested. The tones began at higher sound levels that were gradually lowered from 120 dB to 15 dB. In patients with asymmetric loss, we started with the better ear. The test was performed for all frequencies on one ear first and then the other ear. Pure tone averages (PTA) were computed as the average of the thresholds obtained for the frequencies of 500, 1000, and 2000, according to Davis and Silverman [21].

Also, in an acoustic cabin, we evaluated the patient's ability to recognize speech sounds and measured the Speech Reception Threshold (SRT) for disyllables [22]. SRT is defined as the lowest sound level in which the patient is able to perceive and to repeat out loud correctly 50% of the words presented.

Subjects were also submitted to bone pure tone audiometry in which a pure tone signal is delivered by a bone vibrator (coupled to the arc) placed onto the individuals' mastoid. Hearing thresholds were obtained for the same frequencies used in the air pure tone audiometry. Only patients with sensorineural hearing loss were included, defined as those with equal thresholds measured by air and bone audiometry.

Pure tone audiometry and SRT were also performed in free field. Patients were positioned in an acoustic cabin, this time without headphones [22]. They were instructed to press a button whenever they perceived a sound stimulus. Free-field evaluation allows the calculation of functional gain (FG), a procedure defined by Pascoe [23], and is used to evaluate the efficiency of HA interventions. It consists of computing the percentage change in free field by comparing aided and unaided thresholds, i.e., with and without HA in place.

We first performed the evaluation without HA in place and then with HA positioned in one ear only, while the other ear remained without HA. Functional gain (FG) = aided threshold minus the unaided threshold. Thresholds were obtained for each ear separately. Patients remained seated with one ear pointing to a speaker positioned in the horizontal plane of the ear. First, the tested ear had the HA in place, while the other ear was unaided. Then, HA was removed, and a new threshold was obtained, this time with both ears unaided. The same procedure was repeated with the other ear pointing to the speaker.

Between-group comparison (patients vs. control group) was assessed by the Mann–Whitney *U* test, while within-group differences (patients before HA use × patients after HA use) were inspected by the Wilcoxon test for two dependent samples.

### 2.3. fMRI Acquisition

There were two MRI sessions: right before HA fitting and after one year of HA use. Subjects were scanned in a 1.5 T scanner (Siemens, Magneton Vision, Erlangen, Germany) with a commercially available TX/RX head coil. fMRI acquisition used an echo-planar imaging (EPI) sequence, with the following parameters: 66 volumes, each one composed of 16 axial slices covering both hemispheres, TR = 4600 ms, TE = 60 ms, *flip angle* = 90°, FOV = 220 mm, matrix = 128 × 128, and slice thickness = 5 mm.

Whole brain anatomical T1-weighted images were also acquired using a 3D gradient-recalled echo (GRE) sequence, with the following parameters: TR = 9.7 ms, TE = 4.0 ms, matrix size = 256 × 256, *flip angle* = 12°, FOV = 256 mm, slice number = 154, and slice thickness = 1 mm.

### 2.4. Experimental Paradigm

fMRI auditory stimuli were delivered by MRI compatible headphones (Siemens, Erlangen, Germany) maintaining the same sound level in both ears and for both sessions: before and after HA fitting. The task consisted of listening to a story, presented in a block design, with five blocks of the story (27.5 seconds each) interrupted with five blocks of rest (27.5 seconds each) [24]. The same story was used in both sessions, recorded by a male voice, and delivered to both ears, using the same sound level in both sessions and for all patients (30 dB). Subjects were asked to report the story's content after each session, and story comprehension was rated using a 0–5 Likert scale (0—did not understand at all, 1—understood isolated words, 2—understood 25% of the story, 3—understood 50% of the story, 4—understood 75% of the story, and 5—understood the entire story). Prior to fMRI acquisition, subjects were carefully instructed not to move while in the scanner and to pay as much attention as possible to the story being told.

### 2.5. fMRI Analysis

fMRI data were processed using BrainVoyager QX 1.86 (Brain Innovation, Maastricht, Netherlands) according to the same procedures described elsewhere [24, 25]. Preprocessing steps consisted of motion correction, high pass temporal filter at 0.01 Hz, spatial filtering (FWHM = 4 mm), and transformation into Talairach space. fMRI group differences were analyzed using a fixed-effect general linear model (GLM) with separate subject predictors. Clusters were selected using a threshold corrected for multiple comparisons (*q*[FDR] < 0.05) and with an extension of at least 50 mm^3^. Group analysis included 2 orthogonal contrasts: (i) controls (CG) vs. patients before intervention (PB) and (ii) patients before intervention (PB) vs. patients after intervention (PA).

### 2.6. Correlation Analysis

A *Pearson* correlation analysis was used to assess whether individual fMRI *β*-values were correlated with individual changes in SRT with headphones, computed as a global difference between thresholds observed before and after intervention, according to [SRT (right ear before) + SRT (left ear before)] − [SRT (right ear after) + SRT (left ear after)]. Correlation was computed in specific regions of interest (ROI), involved in the auditory and Wernicke's area (BA22, BA41, and BA42), as well as in brain areas related to multimodal integration, such as the superior temporal sulcus (STS), the middle intraparietal sulcus (IT), and the insula.

### 2.7. Cortical Thickness (CT)

In order to evaluate whether the use of the HA would also be associated with neuroanatomical changes, we used FreeSurfer image analysis suite for cortical reconstruction and volumetric segmentation, which is documented and freely available for download online (http://surfer.nmr.mgh.harvard.edu/). Processing was performed on a Mac-Pro OS X 10.8.2, 2 × 2.26 GHz Quad-Core Intel Xeon. Preprocessing steps included grey/white segmentation, segmentation of the pial surface, for final computation of cortical thickness (CT) maps [26]. Statistical significance was set at *p* < 0.01.

## 3. Results

### 3.1. Audiometric Evaluation

**Figure 1:**
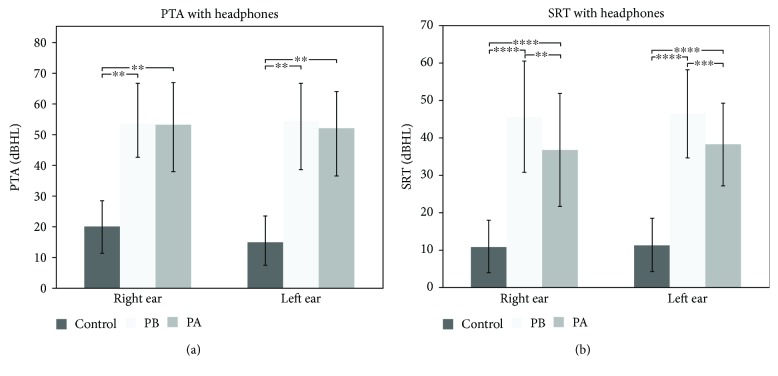
(a) PTA and (b) SRT with headphones of the control group, the patients before HA use (PB), and patients after HA use (PA). (a) Mean and standard deviations are shown for right and left ears. Results of PTA with headphones revealed statistical differences (^∗∗^ *p* < 0.001) in both ears between the CG and PB and PA. When comparing the PB with the PA, statistically significant difference was observed only for the left ear (^∗^ *p* < 0.04). (b) SRT results with headphones demonstrated statistically significant results between PB and PA for the right ear (^∗∗^ *p* < 0.001) and for the left ear (^∗∗∗^ *p* < 0.002). Moreover, statistical analysis indicated significant differences between the GC and PB and PA (^∗^ *p* < 0.0001, for both ears).

Figure 1(a) shows the pure tone averages (PTA) obtained with headphones for all groups. PTA with headphones in the control group (CG) revealed a threshold of 15.68 ± 8.34 dBHL for the right and 14.66 ± 8.47 dBHL for the left ear, which are within the range of normality for adults (0–25 dBHL). Supplementary Figure 1 shows the CG thresholds with headphones for all tested frequencies. Supplementary Table 2 shows individual CG PTA.

Before intervention, PTA with headphones in the patient group was 53.58 ± 12.94 dBHL for the right ear and 54.33 ± 12.10 dBHL for the left ear (Figure 1(a)). After one year of HA use, PTA changed to 53.03 ± 13.61 dBHL and 52.00 ± 11.77 dBHL, respectively, for the right and left ears, which were not significantly different from baseline (Figure 1(a)). We found statistically significant differences between controls and patients before intervention (*p* < 0.001, Figure 1(a)). All patients showed a tonal threshold superior to 25 dBHL for all frequencies tested, both before and after interventions (see Suppl.  Figure 2 and Suppl.  Table 3 for individual results).

Figure 1(b) shows Speech Reception Threshold (SRT) with headphones for all groups studied. The SRT measured with headphones in the CG is considered normal: 10.91 ± 7.01 dBHL and 11.36 ± 7.10 dBHL for the right ear and the left ear, respectively (Figure 1(b)). At baseline, patients showed SRT of 45.71 ± 14.92 and 46.43 ± 11.67 for the right and left ears, respectively (Figure 1(b)). These values reduced significantly after HA use and averaged 36.79 ± 15.14 for the right ear (*p* < 0.001) and 38.21 ± 11.03 (*p* < 0.002) for the left ear (Figure 1(b)). Although a significant improvement was observed, SRT with headphones was still significantly different between controls and patients after HA use, for both ears (*p* < 0.0001, Figure 1(b)). Supplementary Tables 2 and 4 show individual SRT with headphones for all groups studied.

**Figure 2:**
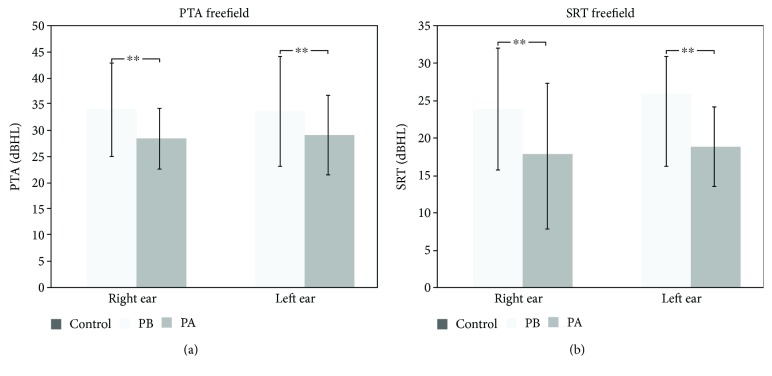
(a) PTA and (b) SRT in free field for the patients before (PB) and after (PA) HA use. Mean and standard deviation are shown for right and left ears before and after HA use. (a) PTA results in free field demonstrated statistically significant changes induced by HA use in both ears (^∗∗^ *p* < 0.001). (b) SRT evaluation in free field also showed a significant improvement in both ears (^∗∗^ *p* < 0.001).

Free-field PTA and SRT were evaluated in patients only (Figure 2). Before HA use, free-field PTA thresholds averaged 33.15 ± 8.48 dBHL (right ear) and 32.68 ± 10.29 dBHL (left ear). After HA use, free-field PTA improved significantly in both ears (*p* < 0.001), reaching 27.68 ± 5.64 dBHL (right ear) and 28.27 ± 7.40 dBHL (left ear). Supplementary Table 5 shows individual free-field PTA, and Supplementary Figure 3 shows free-field tonal audiometry for all frequencies.

Likewise, free-field SRT improved significantly after HA use for both ears (*p* < 0.001, Figure 2(b)). It changed from 24.93 ± 8.36 dBHL (right ear) and 25.71 ± 5.50 dBHL (left ear) to 17.86 ± 8.48 dBHL (right ear) and 18.21 ± 4.64 dBHL (left ear) after HA use (Figure 2(b)). Supplementary Table 6 shows the individual free-field SRT results.

Both PTA and SRT functional gain (FG) improved significantly after HA use. PTA-FG improved significantly for both ears, from 33.15 ± 8.48 dB to 27.68 ± 5.64 dB (right ear, *p* = 0.001) and from 32.68 ± 10.29 dB to 28.27 ± 7.40 dB for the left ear. SRT-FG also showed significant improvement from 23.93 ± 8.36 dB to 17.50 ± 9.15 dB (*p* = 0.001, right ear) and from 25.71 ± 5.49 dB to 18.21 ± 4.64 dB (*p* = 0.001, left ear).

### 3.2. fMRI

Two patients (#5 and #14) had to be excluded from further fMRI analysis due to excessive motion artifact (translation > 2 mm) in at least one of the two sessions, leaving 12 subjects in the final fMRI dataset.

The fMRI task was designed to engage auditory and language receptive fields [25, 27]. Indeed, in control subjects, it produced a robust activation in the auditory cortex for the contrast task (story) vs. baseline in the transverse temporal gyrus (BA41 and BA42) and language centers including Wernicke's area (BA22) (see Suppl.  Figure 4 and Suppl.  Table 7).

**Figure 3:**
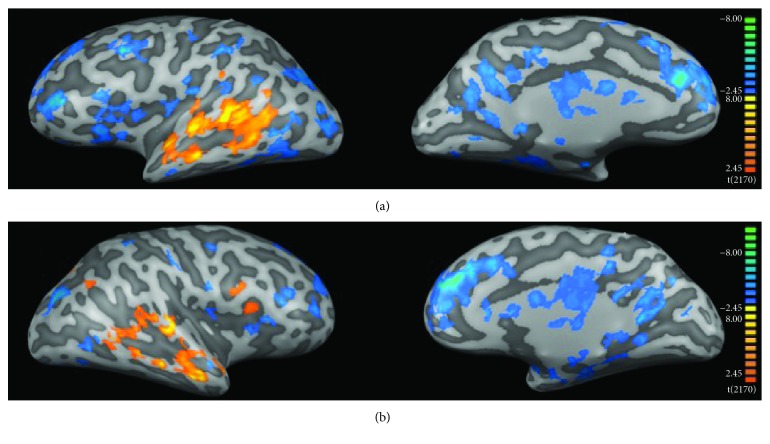
fMRI group analysis: controls versus patients before HA use. (a) Left and (b) right hemispheres, respectively. Color code indicates statistical significance. Warm colors (red-yellowish) show regions where activity was greater in controls than in PB, and cool colors (blue-greenish) show the opposite contrast (PB > CG). Clusters were selected with a *q*[FDR] < 0.05 and size of at least 50 mm^3^.

Figure 3 shows the fMRI results for the comparison between controls and patients before (PB) HA use. Statistical maps were much more diffuse in patients than in controls (Figure 3, Tables 1 and 2). Our results suggest that auditory deprivation is represented by decreased activity in the bilateral auditory cortex (BA41 and BA42) and Wernicke's area (Figure 3, Table 1). We also found increased activity in large portions of the frontal and occipital lobes, including bilateral visual areas (BA17, BA18, and BA19) and areas involved in multimodal integration, such as bilateral superior temporal sulcus (STS), middle intraparietal sulcus (IT, BA40), bilateral inferior frontal gyrus (IFG, BA44, BA45, and BA47), and the insula (BA13) (Figure 3, Table 2).

**Figure 4:**
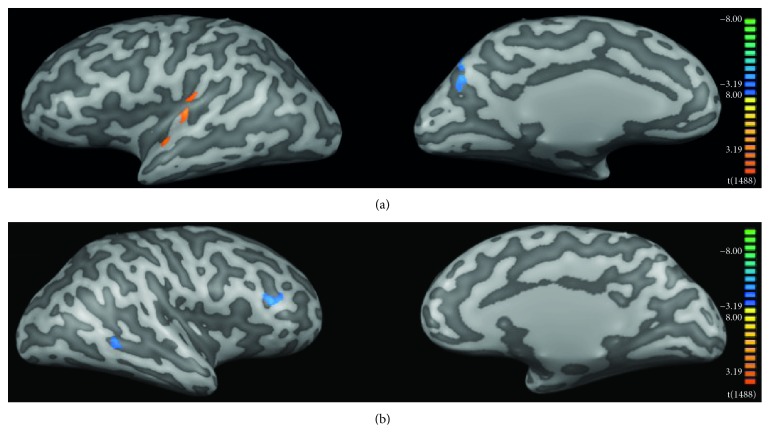
fMRI group analysis: patients before HA use (PB) versus patients after HA use (PA). (a) Left and (b) right hemispheres, respectively. Color code indicates statistical significance. Warm colors (red-yellowish) show regions where activity was greater in PA than in PB, and cool colors (blue-greenish) show the opposite contrast (PB > PA). Clusters were selected with a *q*[FDR] < 0.05 and size of at least 50 mm^3^.

Figure 4 shows the fMRI results from the direct comparison between patients before (PB) vs. after (PA) HA use. Rehabilitation with HA leaded to increased activity of the left transverse temporal gyrus (BA40, BA41), Wernicke's area (left BA22), the left insula (BA13), and left superior frontal gyrus (BA8) (Figure 4, Table 3). We also found that intervention leads to reduced activity in left visual association areas (BA18, BA19), middle and superior frontal gyri (BA9, BA10, and BA46), and the thalamus (Figure 4, Table 4).

**Figure 5:**
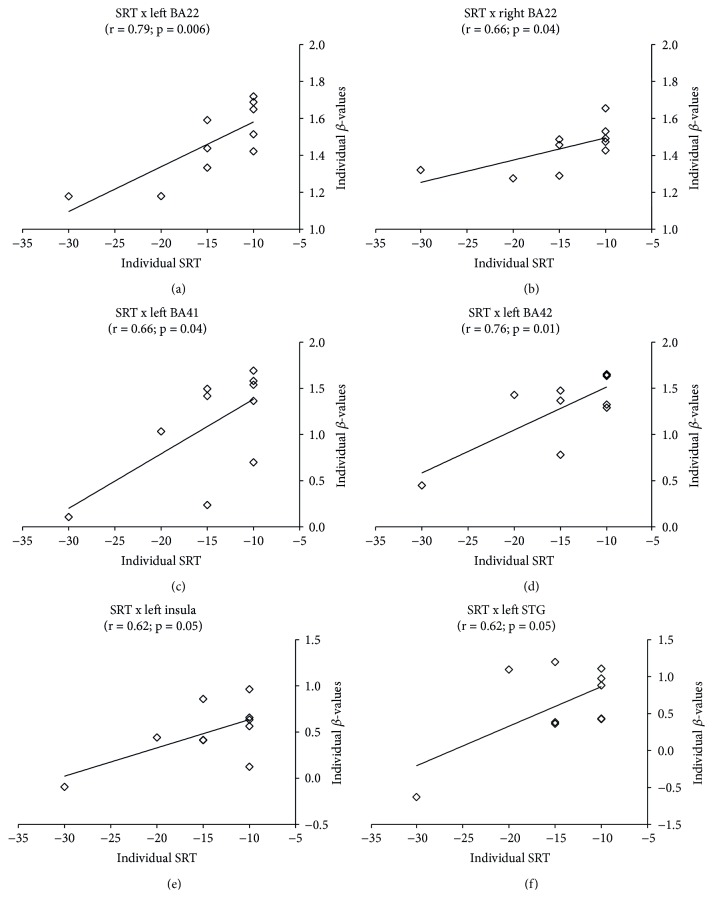
Pearson correlations between individual SRT changes and individual fMRI *β*-values changes. SRT with headphones changes were computed as a global difference between thresholds observed before and after intervention, according to [SRT (right ear before) + SRT (left ear before)] − [SRT (right ear after) + SRT (left ear after)]. Only regions that presented statistically significant correlation are shown. Significant correlations were found only after HA use in (a) left BA22, (b) right BA22, (c) left BA41, (d) left BA42, (e) left insula, and (f) left STG.

Figure 5 shows the correlation between individual changes in fMRI *β*-values and changes in SRT. We observed significant positive correlations in bilateral BA22 (*p* < 0.006, left; *p* < 0.04, right), left BA41 (*p* < 0.04), left BA42 (*p* < 0.01), left insula (*p* < 0.05), and left superior temporal gyrus (*p* < 0.05).

### 3.3. Cortical Thickness Analysis

Cortical thickness (CT) could not be estimated in two patients (#5 and #14) due excessive motion artifact in at least one of the sessions.

**Figure 6:**
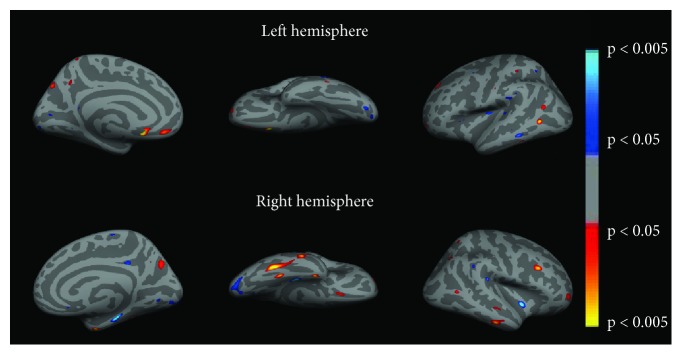
Cortical thickness changes of the patients before HA use (PB) when compared to the control group (CG). Color code indicates *p* values. Warm colors (red-yellowish) show regions where cortical thickness was greater in PB than in the controls, and cool colors (blue-greenish) show the opposite contrast (CG > PB).

Figure 6 shows CT significant differences between controls and patients at baseline (PB). Before intervention, patients presented significant increased CT in bilateral prefrontal cortex (BA9 and BA10), precuneus/superior parietal gyrus (BA7), fusiform gyrus (BA37), and right posterior (BA39) and central portions (BA21) of the middle temporal gyrus (Figure 6, Table 5). We observed reduced CT bilaterally in portions of the visual cortex (BA17 and BA18), insula (BA13), supramarginal gyrus (BA40), left superior (BA41) and middle (BA21) temporal gyri, right parahippocampus (BA35), right posterior cingulate cortex (BA31), and the claustrum (Figure 6, Table 6).

**Figure 7:**
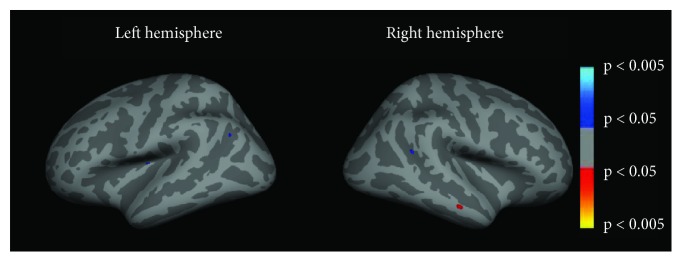
Cortical thickness changes of the patients before HA use (PB) when compared to patients after HA use (PA). Color code indicates *p* values. Warm colors (red-yellowish) show regions where cortical thickness was greater in PB than PA, and cool colors (blue-greenish) show the opposite contrast (PA > PB).

When directly comparing patients before (PB) and after (PA) HA use, cortical thickness was increased in the left angular gyrus (BA39), located at the very caudal end of the superior temporal sulcus and in the right inferior parietal gyrus/superior temporal gyrus/posterior insula (BA13) (Figure 7, Table 7). We did not find areas of significant reduced CT after interventions when compared to baseline values of the patients.

## 4. Discussion

In this study, we explored audiometric, anatomical, and functional brain changes following a one year of continuous HA use in postlingual deaf patients. We observed improved audiometric scores after intervention, particularly of speech recognition, together with fMRI signal increase in the primary auditory cortex, Wernicke's area, and visual areas. HA use also led to decreased fMRI activity in multimodal integration regions, such as the superior temporal sulcus (STS), the middle intraparietal sulcus (IT), and the insula. We observed significant positive correlations between changes in the speech recognition test and increased activity in the primary auditory cortex, Wernicke's area, left insula, and left STS. We also found increased cortical thickness after HA use in the left angular gyrus (BA39) and in the right posterior parietal/temporal junction, including the posterior insula.

Our measured pure tone averages (PTA) suggest that binaural HA fitting in individuals with postlingual sensorineural hearing loss steadies the deterioration of peripheral hearing, as already observed in previous reports [28]. In our study, patients also improved their SRT, both with headphones and in free field. It is well demonstrated that the rehabilitation with HA improves speech recognition, already at six to twelve weeks of HA use [29–31]. We also observed increased functional gain (FG), both for PTA and SRT measurements. Overall, our audiometric results suggest a significant benefit of HA use in speech recognition tasks, while the peripheral auditory system (cochlea, auditory nerve) may not evolve after HA use.

Compared to the control group, patients engaged much broader portions of the brain, including regions in the frontal, parietal, and occipital lobes (Tables 1 and 2). After HA use, activity was reduced in frontal and occipital regions and increased in the auditory cortex, Wernicke's area, and regions involved multimodal integration (Table 4).

Our observations are consistent with previous neuroimaging studies that reported increased activity in auditory-related cortices after CI [13–15]. Besides the auditory system, our results suggest increased activity in the primary and visual association occipital regions (Tables 3 and 4). Increased activity in visual areas has been reported in both fMRI and MEG, in patients with hearing loss [20]. Previous fMRI studies suggest that rehabilitation with CI increases the activity in the left middle occipitotemporal junction (BA37 and BA19) and in the posterior inferior temporal region (BA21 and BA37) [15]. Furthermore, the activity of visual cortex shortly after implantation seems to be related to the level of auditory recovery after cochlear implantation [19], and changes in functional connectivity across visual, temporal, and inferior frontal cortices have important consequences for subsequent CI outcome [32].

Such observations highlight the importance of multimodality as a fundamental aspect of human brain organization. Indeed, the old notion that sensory inputs are processed in specific and unimodal cortices is outdated [33]. For instance, studies in congenitally blind subjects have consistently found increased activity in the primary visual cortex during auditory stimulus processing [34, 35]. Moreover, several lines of evidence indicate that under certain circumstances and for specific visual tasks, hearing impairment leads to increased visual ability following congenital deafness [36]. In our study, we observed augmented fMRI activation of striate cortex (BA17) and extrastriate visual areas (BA18 and BA19), before rehabilitation. Increased recruitment of the visual system of hearing-impaired individuals in response to auditory stimuli has been reported in previous PET studies [37, 38]. Such findings have been interpreted as a result of the increased demand for visual cues during speech processing in individuals with hearing deficits [38]. Possibly as a result of reduced demand, HA use was associated with reduced activity in the secondary and associative visual areas (BA18 and BA19).

Increased activity in frontal areas may reflect increased effort, inner speech with speech production, and/or increased audiovisual (AV) cooperation. In fact, after one year of HA use, we observed significant increased activity in bilateral auditory cortices. Besides, we have found increased activity in Wernicke's area (BA22) (Table 4) and reduced activity in visual areas, such as BA18 and BA19 (Table 3). Together, these results may show a different balance in AV interaction, with a reactivation of auditory speech areas and a more leftward lateralized network, i.e., a more physiological speech processing, less demanding after HA use. The recent study suggests that hearing loss impacts audiovisual speech processing accompanied by changed activity in frontal brain areas, which are modulated by the level of hearing loss [39].

Clinical observations have demonstrated the impact of hearing impairment on higher cognitive processes [2], which can be at least partially recovered by auditory rehabilitation. For instance, it has been observed significant improvements of learning and speech in children after CI [15]. Interestingly, we observed significant correlations between individual fMRI signal changes in auditory (BA41 and BA41) and Wernicke's areas (BA22) and individual change in SRT. This finding links, to our knowledge for the first time, clinical evidence of improved language abilities in patients with hearing loss after auditory rehabilitation with acoustic amplification.

Our results also suggest increased recruitment of brain areas involved in multimodal integration, after HA use, observed as increased fMRI activity in the superior temporal sulcus (STS), the middle intraparietal sulcus (IT, BA40), the inferior frontal gyrus (IFG, BA44, BA45, and BA47), and the insula (BA13). It is possible that HA use improved the quality of information provided by the auditory system to speech integration centers, changing the balance between visual and auditory inputs. In fact, the process of multisensory integration is apparently based upon a weighed estimation of each sensorial input, which in turn depends on the reliability of the information contained in each modality [40]. In further supporting of this hypothesis is the significant positive correlation found between individual fMRI signal changes in the left insula and left STG with individual changes in SRT, such that the greater the SRT improvement, the greater was the fMRI signal change.

The aim of our study was not limited to investigate functional reorganization due to HA use, but it also explored neuroanatomical changes. Before HA use, cortical thickness (CT) was reduced in the visual cortex (BA17 and BA18), primary auditory cortex (BA41), and multimodal cortex (BA13 and BA40) and increased CT was found in the associative somatosensory cortex (BA7), prefrontal cortex (BA9 and BA10), and middle temporal/fusiform gyrus (BA37). Only a few studies have used MRI to investigate neuroanatomical changes due to auditory deprivation, and the results are not consistent. A seminal study used voxel-based morphometry (VBM) in prelingually deaf subjects and identified significant reduced volume only in the left posterior STG [41]. In a more recent study, VBM and CT analysis were applied to evaluate individuals with profound sensorineural hearing loss [42]. No brain structure in the patient group presented increased volume or CT, but the cortical thickness of the primary visual area (BA17) was significantly smaller in patients than in the control group [42]. In another study, CT was investigated in adolescents with prelingual deafness and significant CT differences were found in the right middle occipital gyrus, right precuneus, left gyrus rectus, and left posterior cingulate gyrus [43, 44].

After HA use, our results indicate increased CT at the very caudal end of the STS, including the left angular gyrus (BA39) and the inferior parietal gyrus/superior temporal gyrus/posterior insula (BA13). All of these regions are related to multimodality, and it is tempting to associate these anatomical changes to the functional ones detected by fMRI. Although there are evidence giving support to a possible link between functional and anatomical changes observed by MRI, this is still a matter of debate [45]. Indeed, in some brain areas, the observed increased fMRI activity was related to a reduced CT, as for instance BA17 before HA use. On the other hand, sensory integration areas, such as the left insula, showed increased CT and increased fMRI activity after HA use.

This study has a number of caveats and limitations worth mentioning. First, our sample size was limited to 12 patients in the final functional and anatomical datasets. Second, the absence of a control group (patients without intervention), where patients would be placed on a waiting list for follow-up intervention. However, the nature of this 1-year longitudinal study hinders such design. During audiologic assessments, the nontested ear was not masked or plugged. Therefore, especially in case of mild HL, we might have observed an additive effect between the HA ear and the non-HA ear, and the observed audiometric changes may have been biased by the protocol we used. The same story was presented in both fMRI sessions (before and after HA use), and therefore, our fMRI results are susceptible to habituation. We used a fixed-effects model in the fMRI analysis, which limits our conclusions to the population studied. We did not retest the control group after one year.

To our knowledge, this is the first study which is aimed at investigating audiometric and neuroimaging changes induced by HA use in patients with long-lasting auditory deprivation. Audiometric observations were complemented by neuroimaging investigation, both functional and anatomical cortical thicknesses, to assist in understanding the neurophysiological mechanisms behind hearing rehabilitation. Furthermore, the correlation found between individual fMRI and SRT further paves the perspective for the use of functional neuroimaging as a clinical tool in audiology.

## Figures and Tables

**Table 1:** Brain regions with increased fMRI activity in controls (CG) when compared to patients before HA use (PB). The center of the cluster for each brain region is represented in Talairach coordinates (*x*, *y*, and *z*), followed by its respective standard deviations (in parentheses). Clusters were selected using a *q*[FDR] < 0.05 and a cluster size of at least 50 mm^3^.

Brain region	Hem	Cluster size	Talairach coordinates			BA
			*x*	*y*	*z*	
Middle temporal gyrus	L	2403	−60 (5)	−33 (14)	0 (7)	21, 22, 37, 39
Middle temporal gyrus	R	855	59 (4)	−26 (15)	−3 (6)	21, 22, 37, 39
Transverse temporal gyrus	L	226	−53 (8)	−20 (4)	11 (1)	41, 42
Superior temporal gyrus	L	2893	−56 (6)	−18 (21)	3 (9)	22, 39, 41, 42
Superior temporal gyrus	R	1255	54 (6)	−12 (19)	0 (8)	22, 39, 41, 42
Inferior frontal gyrus	R	278	47 (2)	16 (4)	0 (14)	47
Inferior frontal gyrus	L	107	−51 (3)	15 (5)	0 (12)	47
Middle frontal gyrus	L	223	−2 (1)	−2 (4)	50 (2)	6

Hem = hemisphere; L = left; R = right; and BA = Brodmann area.

**Table 2:** Brain regions with increased fMRI activity in patients before HA use (PB) when compared to controls (CG). The center of the cluster for each brain region is represented in Talairach coordinates (*x*, *y*, and *z*), followed by its respective standard deviations (in parentheses). Clusters were selected using a *q*[FDR] < 0.05 and a cluster size of at least 50 mm^3^.

Brain region	Hem	Cluster size	Talairach coordinates			BA
			*x*	*y*	*z*	
Cuneus	R	601	14 (5)	−78 (6)	11 (4)	17, 18
Lingual gyrus	R	872	20 (7)	−73 (11)	−2 (5)	17, 18, 19
Lingual gyrus	L	930	−19 (6)	−66 (11)	−3 (5)	17, 18, 19
Precuneus	R	355	12 (7)	−61 (8)	25 (5)	19
Precuneus	L	392	−13 (11)	−59 (8)	29 (7)	19
Fusiform gyrus	R	489	33 (7)	−60 (12)	−12 (3)	19, 37
Fusiform gyrus	L	770	−32 (9)	−62 (19)	−13 (3)	18, 19, 37
Middle occipital gyrus	L	565	−33 (8)	−80 (8)	3 (8)	18, 19
Superior temporal gyrus	L	178	−45 (5)	−43 (13)	19 (8)	13, 22, 41, 39
Superior temporal gyrus	R	152	43 (7)	−52 (5)	20 (3)	13, 22, 39
Inferior temporal gyrus	L	318	−53 (5)	−38 (27)	−10 (9)	19, 20
Middle temporal gyrus	L	374	−49 (10)	−45 (25)	2 (11)	19, 21
Parahippocampal gyrus	R	1131	24 (6)	−22 (14)	−14 (7)	28, 34, 35, 36, hippocampus, amygdala
Parahippocampal gyrus	L	1237	−25 (8)	−26 (12)	−12 (7)	27, 28, 34, 35, 36, hippocampus, amygdala
Cingulate gyrus	L	1206	−7 (5)	−2 (28)	34 (5)	23, 24, 31, 32
Cingulate gyrus	R	2492	7 (4)	−2 (22)	34 (5)	23, 24, 30, 31, 32
Anterior cingulate	L	482	−10 (5)	37 (3)	18 (5)	32
Anterior cingulate	R	935	8 (5)	35 (9)	15 (7)	24, 32, 33
Posterior cingulate	L	873	−6 (6)	−54 (6)	17 (5)	23, 29, 30, 31
Posterior cingulate	R	974	8 (7)	−54 (9)	15 (5)	23, 29, 30, 31
Insula	R	616	37 (4)	4 (18)	12 (6)	13
Insula	L	1213	−38 (5)	−5 (19)	12 (8)	13
Inferior frontal gyrus	L	796	−45 (8)	16 (6)	10 (13)	6, 9, 10, 44, 45, 46, 47
Middle frontal gyrus	L	2596	−6 (5)	39 (11)	28 (10)	6, 8, 9, 10
Middle frontal gyrus	R	2607	7 (4)	41 (10)	26 (11)	6, 8, 9, 10
Middle frontal gyrus	R	242	38 (7)	23 (20)	27 (12)	6, 9, 10, 46
Middle frontal gyrus	L	1416	−37 (8)	26 (20)	29 (14)	6, 8, 9, 10, 46
Superior frontal gyrus	R	882	11 (6)	53 (5)	29 (5)	8, 9, 10
Superior frontal gyrus	L	1932	−15 (10)	48 (13)	32 (10)	6, 8, 9, 10
Precentral gyrus	L	465	−44 (5)	2 (6)	32 (11)	4, 6, 9, 43
Precentral gyrus	R	497	45 (6)	−7 (7)	34 (9)	4, 6
Inferior parietal lobe	L	467	−44 (6)	−37 (7)	38 (7)	39, 40
Caudate	L	876	−14 (7)	−6 (16)	16 (6)	
Caudate	R	938	18 (6)	−11 (17)	17 (6)	
Thalamus	L	489	−7 (5)	−16 (8)	9 (5)	
Thalamus	R	1144	16 (6)	−17 (7)	10 (4)	

Hem = hemisphere; L = left; R = right; and BA = Brodmann area.

**Table 3:** Brain regions with increased fMRI activity in patients before HA use (PB) when compared to patients after HA use (PA). The center of the cluster for each brain region is represented in Talairach coordinates (*x*, *y*, and *z*), followed by its respective standard deviations (in parentheses). Clusters were selected using a *q*[FDR] < 0.05 and a cluster size of at least 50 mm^3^.

Brain region	Hem	Cluster size	Talairach coordinates			BA
			*x*	*y*	*z*	
Cuneus	L	118	−12 (2)	−76 (2)	32 (1)	18, 19
Precuneus	L	156	−15 (2)	−73 (5)	33 (6)	19
Middle frontal gyrus	R	260	43 (3)	37 (5)	17 (2)	10, 46
Middle frontal gyrus	R	89	6 (2)	47 (1)	28 (2)	9
Superior frontal gyrus	R	70	6 (2)	49 (1)	30 (2)	9
Superior frontal gyrus	L	102	−4 (1)	55 (2)	25 (2)	9
Thalamus	R	325	12 (3)	−22 (3)	14 (2)	

Hem = hemisphere; L = left; R = right; and BA = Brodmann area.

**Table 4:** Brain regions with increased fMRI activity in patients after HA use (PA) when compared to patients before HA use (PB). The center of the cluster for each brain region is represented in Talairach coordinates (*x*, *y*, and *z*), followed by its respective standard deviations (in parentheses). Clusters were selected using a *q*[FDR] < 0.05 and a cluster size of at least 50 mm^3^.

Brain region	Hem	Cluster size	Talairach coordinates			BA
			*x*	*y*	*z*	
Superior temporal gyrus	L	476	−51 (5)	−1 (10)	1 (4)	21, 22, 41
Transverse temporal gyrus	L	178	−42 (4)	−23 (2)	12 (1)	40, 41
Superior frontal gyrus	L	295	−6 (2)	40 (4)	46 (3)	8
Insula	L	109	−39 (4)	−23 (7)	12 (4)	13

Hem = hemisphere; L = left; R = right; and BA = Brodmann area.

**Table 5:** Regions of increased cortical thickness in patients at baseline (PB) when compared to controls (CG). Mean cortical thickness is expressed in mm. The numbers in parentheses correspond to standard deviations. Statistical significance was based at *p* < 0.01.

Brain region	Hem	Nvox	Talairach coordinates			BA	PB	CG	*p* value
			*x*	*y*	*z*		Mean (SD)	Mean (SD)	
Medial orbitofrontal gyrus	L	176	−9.0	37.8	−13.5	10	3.12 (0.36)	2.54 (0.35)	0.001
Middle frontal gyrus	L	111	−21.2	52.3	−9.7	10	2.83 (0.41)	2.45 (0.20)	0.010
Middle frontal gyrus	R	226	38.9	20.0	25.7	9	2.89 (0.22)	2.59 (0.21)	0.005
Superior parietal gyrus	L	209	−16.6	−70.1	37.3	7	2.35 (0.29)	2.00 (0.30)	0.010
Superior parietal gyrus	R	55	22.6	−53.5	57.5	7	2.28 (0.16)	2.01 (0.28)	0.010
Precuneus	L	58	−7.8	−52.7	37.5	7	2.96 (0.37)	2.50 (0.40)	0.010
Precuneus	R	279	18.8	−66.1	34.2	7	2.49 (0.29)	2.21 (0.16)	0.010
Fusiform gyrus	R	626	40.7	−48.7	−11.0	37	3.28 (0.16)	2.84 (0.25)	0.0002
Fusiform gyrus	L	127	−50.9	−58.3	3.3	37	3.08 (0.21)	2.59 (0.29)	0.0003
Middle temporal gyrus	R	281	54.5	−20.4	−18.6	21	3.34 (0.26)	2.95 (0.30)	0.005
Middle temporal gyrus	R	56	49.4	−59.1	7.4	39	3.11 (0.21)	2.73 (0.38)	0.010
Entorhinal gyrus	R	161	30.2	−3.5	−29.0	36	3.94 (0.50)	3.33 (0.31)	0.003

Hem = hemisphere; L = left; R = right; BA = Brodmann area; Nvox = number of voxels in the cluster; SD = standard deviation; PB = patients before HA use; CG = control group.

**Table 6:** Regions of reduced cortical thickness in patients at baseline (PB) when compared to controls (CG). Mean cortical thickness is expressed in mm. The numbers in parentheses correspond to standard deviations. Statistical significance was set at *p* < 0.01.

Brain region	Hem.	Nvox	Talairach coordinates			BA	PB	CG	*p* value
			*x*	*y*	*z*		Mean (SD)	Mean (SD)	
Insula	R	144	44.5	−35.3	19.9	13	2.54 (0.22)	2.87 (0.21)	0.003
Insula	L	166	−34.5	−14.9	13.5	13	2.93 (0.24)	3.35 (0.35)	0.005
Supramarginal gyrus	R	64	52.6	−39.3	30.6	40	2.66 (0.27)	3.09 (0.37)	0.007
Supramarginal gyrus	L	100	−55.8	−29.1	21.9	40	2.76 (0.34)	3.13 (0.29)	0.010
Superior temporal gyrus	L	55	−42.6	−28.7	5.0	41	2.71 (0.40)	3.34 (0.61)	0.010
Middle temporal gyrus	L	116	−58.4	−38.2	−9.3	21	2.92 (0.47)	3.61 (0.33)	0.001
Parahippocampal gyrus	R	336	23.8	−24.1	−19.0	35	3.06 (0.20)	3.43 (0.30)	0.004
Lateral occipital gyrus	R	462	21.5	−89.6	−2.2	17	2.13 (0.29)	2.55 (0.30)	0.004
Lingual gyrus	R	45	8.7	−69.9	3.9	18	2.15 (0.29)	2.55 (0.39)	0.010
Middle occipital gyrus	L	71	−23.1	−82.8	−6.5	18	2.19 (0.32)	2.69 (0.37)	0.004
Posterior cingulate	R	99	8.4	−34.9	33.0	31	3.00 (0.42)	3.67 (0.55)	0.006
Claustrum	R	187	35.5	−4.0	−4.6	—	3.40 (0.53)	4.16 (0.40)	0.001

Hem = hemisphere; L = left; R = right; BA = Brodmann area; Nvox = number of voxels in the cluster; SD = standard deviation; PB = patients before HA use; CG = control group.

**Table 7:** Regions of increased cortical thickness in the patients after HA use (PA) when compared to patients before HA use (PB). Mean cortical thickness is expressed in mm. The numbers in parentheses correspond to standard deviations. Statistical significance was based at *p* < 0.01.

Brain region	Hem	Nvox	Talairach coordinates			BA	PB	PA	*p* value
			*x*	*y*	*z*		Mean (SD)	Mean (SD)	
Inferior parietal gyrus/superior temporal gyrus/posterior insula	R	36	44.7	−44.5	18.8	13	2.57 (0.28)	2.97 (0.29)	0.010
Angular gyrus	L	31	−38.6	−58.9	29.9	39	2.73 (0.37)	3.06 (0.29)	0.003

Hem = hemisphere; L = left; R = right; BA = Brodmann area; Nvox = number of voxels in the cluster; SD = standard deviation; PB = patients before HA use; PA = patients after HA use.

## Data Availability

The data used to support the findings of this study are available from the corresponding author upon request.

## Supplementary Materials

Supplementary Materials Supplementary Figure 1: pure tone audiometry for each frequency tested with headphones in the control group. Supplementary Figure 2: pure tone audiometry for each frequency tested with headphones for the group of patients before (PB) and after (PA) HA use. Supplementary Figure 3: pure tone audiometry for each frequency tested in free field, for patients before (PB) and after (PA) HA use. Supplementary Figure 4: fMRI statistical maps of the control group. Supplementary Table 1: clinical and audiometric information of the patients. Supplementary Table 2: individual PTA and SRT with headphones for the control group (CG). Supplementary Table 3: individual PTA with headphones for patients before (PB) and after (PA) HA use. Supplementary Table 4: individual SRT with headphones of the group of patients before (PB) and after (PA) HA use. Supplementary Table 5: individual PTA in free field of the group of patients before (PB) and after (PA) HA use. Supplementary Table 6: individual SRT in free field for patients before (PB) and after (PA) HA use. Supplementary Table 7: statistically significant fMRI response in the control group.
