# Supplementary material for: Anatomical and Functional MRI Changes after One Year of Auditory Rehabilitation with Hearing Aids
Source: Neural Plast. 2018 Sep 10;2018:9303674. doi: 10.1155/2018/9303674 (PMC6151682; doi:10.1155/2018/9303674)
Supplement: Supplementary Materials — Supplementary Figure 1: pure tone audiometry for each frequency tested with headphones in the control group. Supplementary Figure 2: pure tone audiometry for each frequency tested with headphones for the group of patients before (PB) and after (PA) HA use. Supplementary Figure 3: pure tone audiometry for each frequency tested in free field, for patients before (PB) and after (PA) HA use. Supplementary Figure 4: fMRI statistical maps of the control group. Supplementary Table 1: clinical and audiometric information of the patients. Supplementary Table 2: individual PTA and SRT with headphones for the control group (CG). Supplementary Table 3: individual PTA with headphones for patients before (PB) and after (PA) HA use. Supplementary Table 4: individual SRT with headphones of the group of patients before (PB) and after (PA) HA use. Supplementary Table 5: individual PTA in free field of the group of patients before (PB) and after (PA) HA use. Supplementary Table 6: individual SRT in free field for patients before (PB) and after (PA) HA use. Supplementary Table 7: statistically significant fMRI response in the control group. [file 9303674.f1.docx]

**Supplementary Material**

**Anatomical and functional MRI changes after one-year of auditory rehabilitation with hearing aids**

**Supplementary figure 1.** **Pure tone audiometry for each frequency tested with headphones in the control group.** Average and standard deviation thresholds with headphone for right (
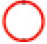
) and left (
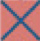
) ears tested at different frequencies.

**
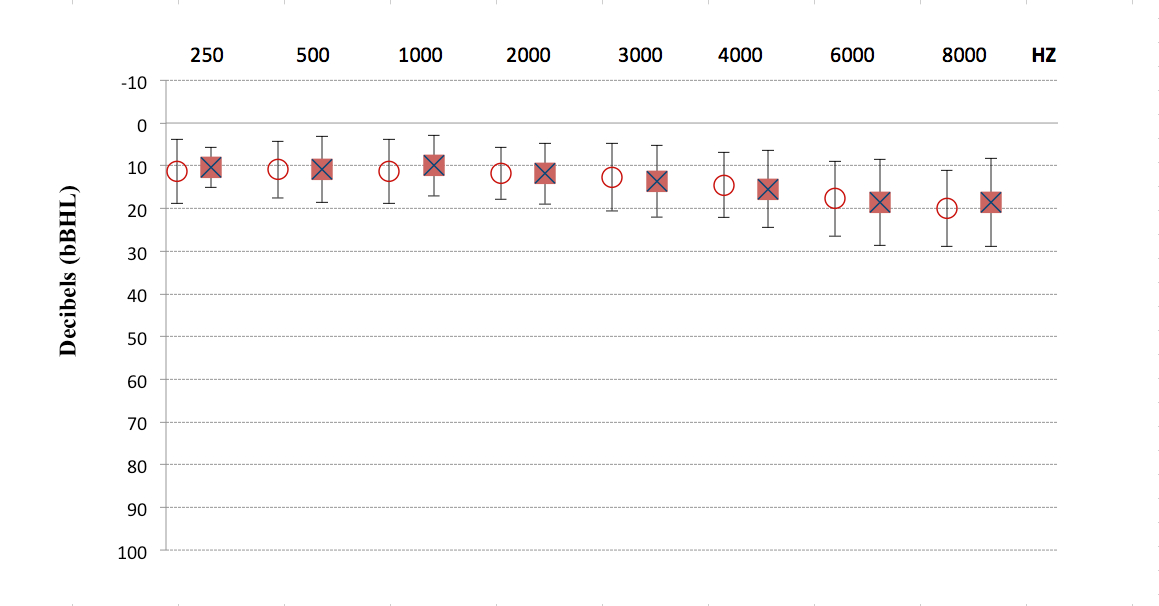
**

**Supplementary figure 2. Pure tone audiometry for each frequency tested with headphones for the group of patients before (PB) and after (PA) HA use.** Mean and standard deviation are shown for right (
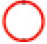
) and left (
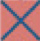
) ears.

**Before HA use**

**
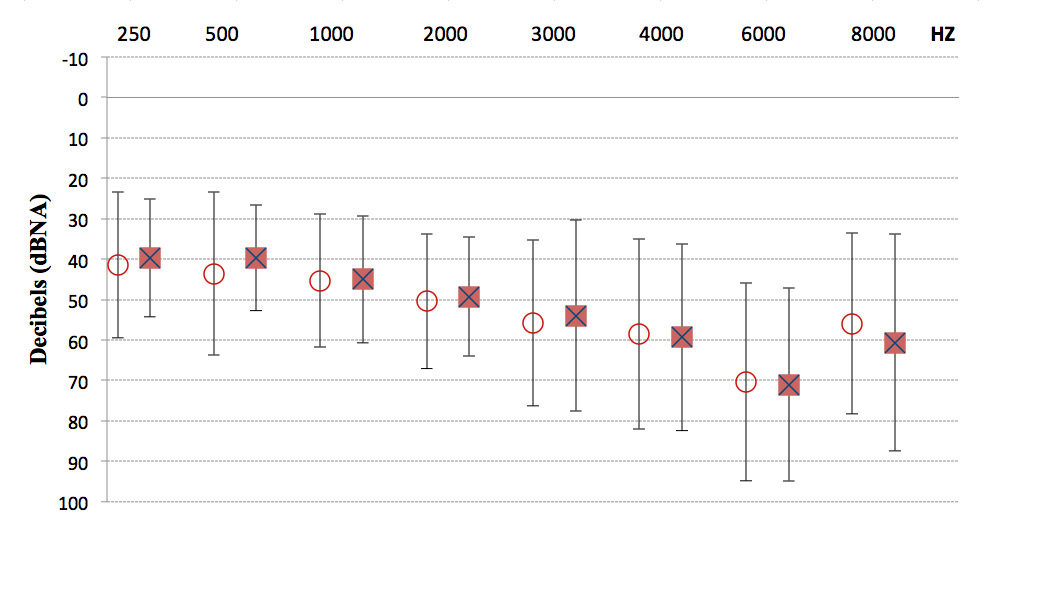
**

**(a)**

**After HA use**

**
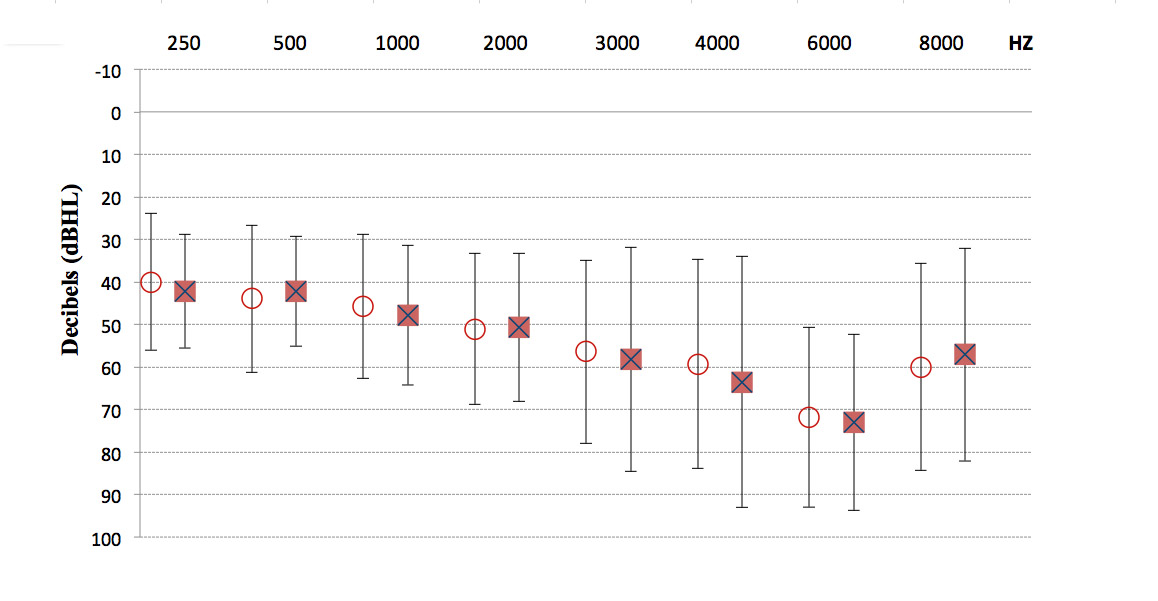
**

**(b)**

**Supplementary figure 3. Pure tone audiometry for each frequency tested in free field, for patients before (PB) and after (PA) HA use.** Mean and standard deviation are shown for **(a)** right and **(b)** left ears. Statistically significant differences between the PB and PA were observed in every frequency tested. *p = 0.001.

Tonal audiometry

**
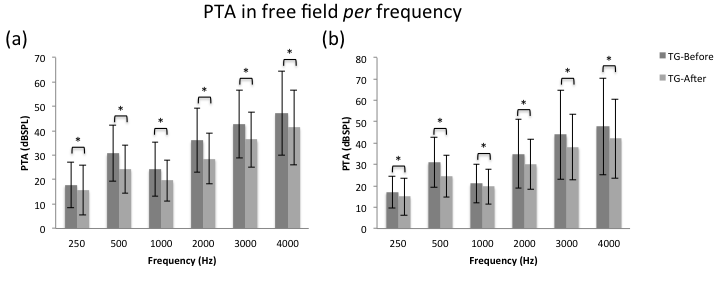
**

PB

PA

**Supplementary figure 4. fMRI statistical maps of the control group.** (a) and (b) show the left and right hemisphere respectively. Color code indicates t values (q[FDR] < 0.05 and cluster size of at least 50 mm^3^).


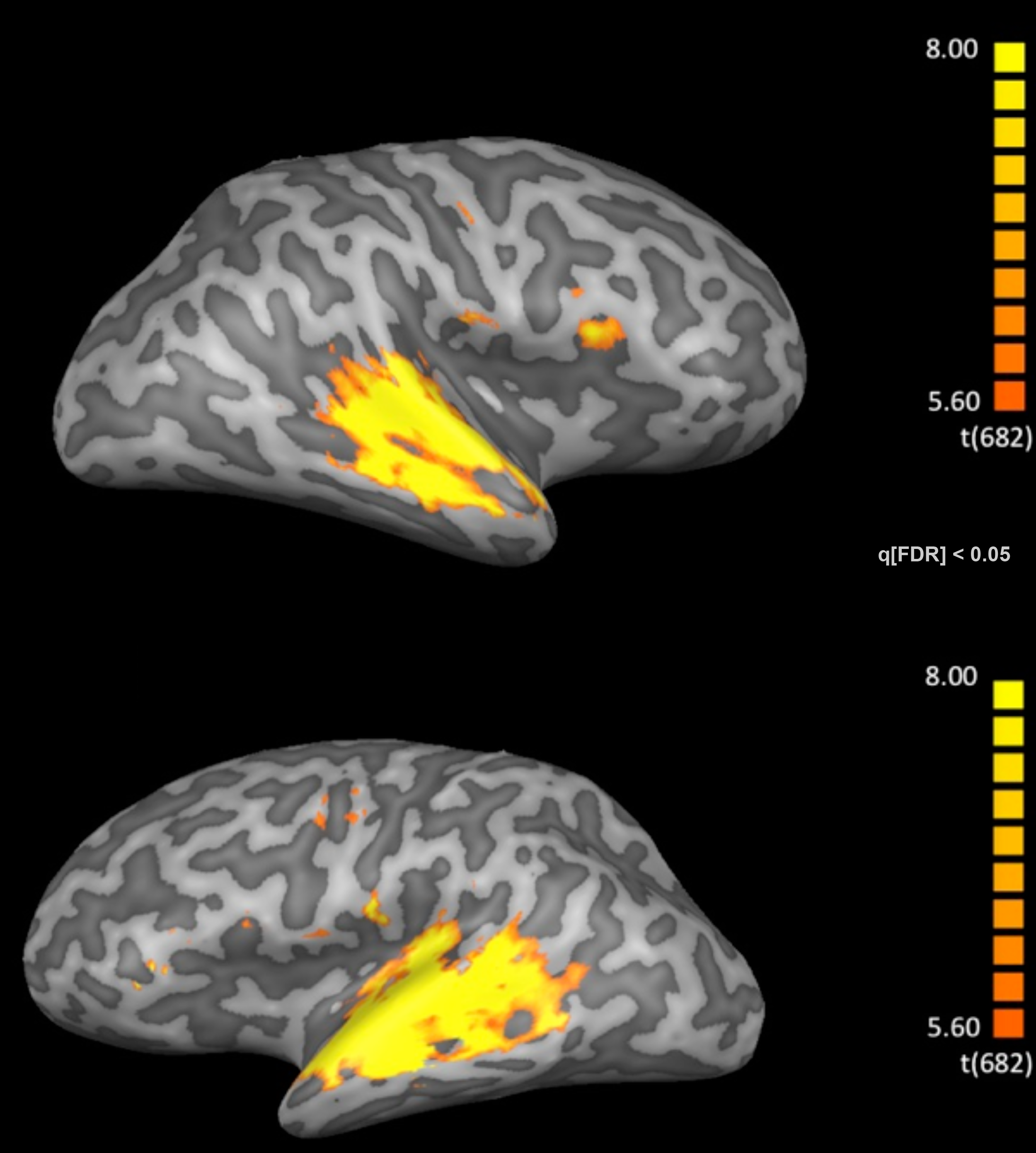


(b)

(a)

**Supplementary table 1.** **Clinical and audiometric information of the patients.**

| **Subject** | | **Age** | **Gender** | **Duration of HL** | **HA type** | **Degree of hearing loss** | | **Etiology** |
| --- | --- | --- | --- | --- | --- | --- | --- | --- |
|  |  |  |  |  |  | **Right** | **Left** |  |
| 1 | | 37 | F | 32 years | ITC | moderate | moderate | Congenital |
| 2 | | 19 | M | 9 years | ITC | moderate | moderate | Infection |
| 3 | | 74 | M | 15 years | CIC | moderate | moderate | Genetic |
| 4 | | 29 | F | 19 years | CIC | mild | mild | Genetic |
| 5 | | 42 | M | 8 years | ITC | moderate | moderate | Unknown |
| 6 | | 59 | M | 10 years | ITC | moderate | moderate | Presbycusis |
| 7 | | 61 | M | 15 years | ITC | moderate | moderate | noise-induced hearing loss |
| 8 | | 55 | M | 10 years | CIC | moderate | moderate | noise-induced hearing loss |
| 9 | | 61 | F | 15 years | ITC | moderate | moderate | Unknown |
| 10 | | 27 | F | 10 years | CIC | mild | mild | Genetic |
| 11 | | 43 | M | 9 years | ITC | moderate | severe | Unknown |
| 12 | | 62 | M | 8 years | ITC | moderate | moderate | Presbycusis |
| 13 | | 67 | M | 7 years | ITC | moderate | severe | Presbycusis |
| 14 | | 82 | F | 12 years | ITC | severe | moderate | Presbycusis |
|  | Completely-In-Canal (CIC); In-The-Canal (ITC); HL: hearing loss. | | | | | | | |

**Supplementary table 2. Individual PTA and SRT with headphones for the control group (CG).**

| **Subject** | **Gender** | **Age** | **PTA (dBHL)** | | **SRT (dBHL)** | |
| --- | --- | --- | --- | --- | --- | --- |
|  |  |  | **Right** | **Left** | **Right** | **Left** |
| 1 | M | 42 | 14.38 | 16.25 | 15 | 15 |
| 2 | F | 72 | 19.38 | 20.00 | 10 | 15 |
| 3 | M | 78 | 33.75 | 33.13 | 25 | 25 |
| 4 | M | 22 | 13.13 | 10.63 | 5 | 5 |
| 5 | F | 39 | 5.63 | 6.25 | 5 | 5 |
| 6 | M | 18 | 6.25 | 6.25 | 5 | 5 |
| 7 | M | 36 | 11.88 | 8.75 | 10 | 10 |
| 8 | F | 41 | 8.75 | 6.25 | 5 | 5 |
| 9 | F | 37 | 15.00 | 15.00 | 5 | 5 |
| 10 | F | 66 | 24.38 | 23.75 | 20 | 20 |
| 11 | F | 61 | 20.00 | 15.00 | 15 | 15 |
| **Average** |  | **46.55** | **15.68** | **14.66** | **10.91** | **11.36** |
| **Standard Deviation** |  | **19.88** | **8.34** | **8.47** | **7.01** | **7.10** |

**Supplementary table 3. Individual PTA with headphone for patients before (PB) and after (PA) HA use.**

| \|  \| **PTA (dBHL)** \| \| \| \| \| --- \| --- \| --- \| --- \| --- \| \| **PB** \| \| **PA** \| \| \| **Subject** \| **Right** \| **Left** \| **Right** \| **Left** \| \| 1 \| 62.86 \| 65.00 \| 64.29 \| 63.57 \| \| 2 \| 62.86 \| 46.43 \| 67.86 \| 47.86 \| \| 3 \| 51.25 \| 49.29 \| 50.63 \| 51.88 \| \| 4 \| 30.00 \| 33.13 \| 30.63 \| 30.00 \| \| 5 \| 60.00 \| 62.50 \| 56.25 \| 58.75 \| \| 6 \| 57.50 \| 52.14 \| 55.00 \| 50.00 \| \| 7 \| 45.00 \| 57.14 \| 46.25 \| 55.71 \| \| 8 \| 53.75 \| 56.88 \| 51.88 \| 52.50 \| \| 9 \| 49.38 \| 47.50 \| 50.63 \| 46.25 \| \| 10 \| 28.75 \| 33.13 \| 28.75 \| 31.88 \| \| 11 \| 66.25 \| 72.50 \| 68.75 \| 75.63 \| \| 12 \| 54.38 \| 55.00 \| 48.13 \| 51.88 \| \| 13 \| 51.88 \| 72.50 \| 46.25 \| 62.14 \| \| 14 \| 76.25 \| 57.50 \| 77.14 \| 50.00 \| \| **Average** \| **53.58** \| **54.33** \| **53.03** \| **52.00** \| \| **Standard Deviation** \| **12.94** \| **12.10** \| **13.61** \| **11.77** \| |
| --- | --- | --- | --- | --- | --- | --- | --- | --- | --- | --- | --- | --- | --- | --- | --- | --- | --- | --- | --- | --- | --- | --- | --- | --- | --- | --- | --- | --- | --- | --- | --- | --- | --- | --- | --- | --- | --- | --- | --- | --- | --- | --- | --- | --- | --- | --- | --- | --- | --- | --- | --- | --- | --- | --- | --- | --- | --- | --- | --- | --- | --- | --- | --- | --- | --- | --- | --- | --- | --- | --- | --- | --- | --- | --- | --- | --- | --- | --- | --- | --- | --- | --- | --- | --- | --- | --- | --- | --- | --- | --- | --- | --- | --- | --- |

PB: patient before HA use; PA: patient after HA use**Supplementary table 4. Individual SRT with headphones of the group of patients before (PB) and after (PA) HA use.** The global difference was calculated according to: [SRT (right ear before HA) + SRT (left ear before HA)] – [SRT (right ear after HA) + SRT (left ear after HA)]. The comprehension of the story used in the fMRI paradigm received a ratting from 0 – 5: 0 – didn’t understand at all; 1 – understood isolated words; 2 – understood 25 % of the story; 3 – understood 50 % of the story; 4 – understood 75 % of the story; 5 – understood the entire story.

|  | **SRT (dBHL)** | | | | | | | **story comprehension** | |
| --- | --- | --- | --- | --- | --- | --- | --- | --- | --- |
|  | **Before** | | **After** | | **Difference** | | **Global Difference** | **Before HA** | **After HA** |
| **Subject** | **Right** | **Left** | **Right** | **Left** | **Right** | **Left** |  |  |  |
| 1 | 55 | 55 | 45 | 50 | -10 | -5 | -15 | 1 | 2 |
| 2 | 55 | 35 | 50 | 30 | -5 | -5 | -10 | 3 | 5 |
| 3 | 35 | 30 | 25 | 25 | -10 | -5 | -15 | 3 | 5 |
| 4 | 35 | 30 | 25 | 30 | -10 | 0 | -10 | 3 | 5 |
| 5 | 55 | 50 | 40 | 35 | -15 | -15 | -15 | 3 | 5 |
| 6 | 50 | 55 | 40 | 50 | -10 | -5 | -20 | 3 | 4 |
| 7 | 20 | 40 | 10 | 30 | -10 | -10 | -10 | 3 | 5 |
| 8 | 35 | 35 | 30 | 30 | -5 | -5 | -10 | 3 | 5 |
| 9 | 45 | 45 | 40 | 40 | -5 | -5 | -30 | 2 | 3 |
| 10 | 50 | 45 | 35 | 30 | -15 | -15 | -10 | 3 | 5 |
| 11 | 65 | 65 | 60 | 60 | -5 | -5 | -15 | 1 | 3 |
| 12 | 35 | 45 | 30 | 35 | -5 | -10 | -10 | 3 | 5 |
| 13 | 30 | 65 | 20 | 55 | -10 | -10 | -15 | 3 | 4 |
| 14 | 75 | 55 | 65 | 35 | -10 | -20 | -10 | 1 | 2 |
| **Average** | **46** | **46** | **37** | **38** | **-9** | **-8** | **-14** | **3** | **4** |
| **Standard Deviation** | **15** | **12** | **15** | **11** | **3** | **5** | **6** | **1** | **1** |

**Supplementary table 5. Individual PTA in free field of the group of patients before (PB) and after (PA) HA use.**

| **Subject** | **PTA (dBHL)** | | | |
| --- | --- | --- | --- | --- |
|  | **Before** | | **After** | |
|  | **Right** | **Left** | **Right** | **Left** |
| 1 | 46.67 | 47.50 | 37.50 | 35.83 |
| 2 | 34.17 | 25.83 | 26.67 | 21.67 |
| 3 | 30.83 | 30.83 | 28.33 | 30.00 |
| 4 | 22.50 | 20.00 | 20.83 | 19.17 |
| 5 | 36.67 | 41.67 | 34.17 | 36.67 |
| 6 | 33.33 | 30.00 | 27.50 | 30.00 |
| 7 | 23.33 | 21.67 | 21.67 | 20.00 |
| 8 | 26.67 | 29.17 | 25.00 | 25.00 |
| 9 | 29.17 | 27.50 | 26.67 | 25.00 |
| 10 | 20.83 | 25.00 | 21.67 | 23.33 |
| 11 | 45.00 | 50.00 | 30.00 | 39.17 |
| 12 | 32.50 | 31.67 | 22.50 | 25.83 |
| 13 | 36.67 | 50.00 | 26.67 | 41.67 |
| 14 | 45.83 | 26.67 | 38.33 | 22.50 |
| **Average** | **33.15** | **32.68** | **27.68** | **28.27** |
| **Standard Deviation** | **8.48** | **10.29** | **5.64** | **7.40** |

**Supplementary table 6. Individual SRT in free field for patients before (PB) and after (PA) HA use.**

| **Subject** | **SRT (dBHL)** | | | |
| --- | --- | --- | --- | --- |
|  | **Before** | | **After** | |
|  | **Right** | **Left** | **Right** | **Left** |
| 1 | 25 | 30 | 20 | 20 |
| 2 | 30 | 20 | 20 | 10 |
| 3 | 20 | 25 | 10 | 20 |
| 4 | 20 | 20 | 15 | 15 |
| 5 | 35 | 35 | 25 | 25 |
| 6 | 20 | 30 | 15 | 20 |
| 7 | 10 | 15 | 5 | 10 |
| 8 | 15 | 20 | 10 | 15 |
| 9 | 20 | 25 | 15 | 20 |
| 10 | 25 | 30 | 25 | 20 |
| 11 | 35 | 30 | 30 | 25 |
| 12 | 20 | 25 | 15 | 20 |
| 13 | 20 | 30 | 10 | 20 |
| 14 | 40 | 25 | 35 | 15 |
| **Average** | **24.93** | **25.71** | **17.86** | **18.21** |
| **Standard Deviation** | **8.36** | **5.50** | **8.48** | **4.64** |

**Supplementary table 7. Statistically significant fMRI response in the control group.** Talairach coordinates of the center of the cluster for each brain region are represented by (x, y, z), followed by its respective standard deviations (in parenthesis). Clusters were selected using a q[FDR] < 0.05 and a cluster size of at least 50 mm^3^. Hem = Hemisphere, L = left, R = right and BA = Brodmann area.

| **Brain Region** | **Hem** | **Cluster size** | **Talairach coordinates** | | | **BA** |
| --- | --- | --- | --- | --- | --- | --- |
|  |  |  | **x** | **y** | **z** |  |
| Superior Temporal Gyrus | L | 4544 | -53 (7) | -14 (15) | 1 (6) | 21, 22, 41, 42 |
| Superior Temporal Gyrus | R | 3798 | 54 (6) | -11 (14) | 1 (6) | 21, 22, 41, 42 |
| Insula | L | 595 | -39 (3) | -19 (4) | 5 (5) | 13 |
| Insula | R | 549 | 42 (4) | -18 (1) | 4 (4) | 13 |
| Transverse Temporal Gyrus | L | 694 | -49 (8) | -21 (6) | 11 (1) | 41, 42 |
| Transverse Temporal Gyrus | R | 616 | 53 (7) | -19 (5) | 11 (1) | 41, 42 |
| Middle Temporal Gyrus | L | 2409 | -59 (4) | -26 (14) | -1 (5) | 21, 22 |
| Middle Temporal Gyrus | R | 1296 | 59 (3) | -21 (10) | -2 (4) | 21, 22 |
| Precentral Gyrus | L | 206 | -47 (5) | -6 (2) | 35 (17) | 4, 6 |
| Precentral Gyrus | R | 312 | 54 (5) | -6 (4) | 22 (18) | 4, 6 |
| Inferior Frontal Gyrus | L | 340 | 48 (2) | 16 (3) | 4 (15) | 47 |
